# Dopant-Free Hole-Transporting Material Based on Poly(2,7-(9,9-bis(N,N-di-p-methoxylphenylamine)-4-phenyl))-fluorene for High-Performance Air-Processed Inverted Perovskite Solar Cells

**DOI:** 10.3390/polym15122750

**Published:** 2023-06-20

**Authors:** Baomin Zhao, Meng Tian, Xingsheng Chu, Peng Xu, Jie Yao, Pingping Hou, Zhaoning Li, Hongyan Huang

**Affiliations:** 1 State Key Laboratory for Organic Electronics and Information Displays & Jiangsu Key Laboratory for Biosensors, Institute of Advanced Materials (IAM), Nanjing University of Posts and Telecommunications, 9 Wenyuan Road, Nanjing 210023, China; 2 School of Electronic Information, Nanjing Vocational College of Information Technology, 99 Wenyuan Road, Nanjing 210023, China

**Keywords:** fluorene-based hole transporting polymer, dopant-free, air-processed, inverted perovskite solar cells

## Abstract

It is a great challenge to develop low-cost and dopant-free polymer hole-transporting materials (HTM) for PSCs, especially for efficient air-processed inverted (p-i-n) planar PSCs. A new homopolymer HTM, poly(2,7-(9,9-bis(N,N-di-p-methoxylphenyl amine)-4-phenyl))-fluorene (denoted as PFTPA), with appropriate photo-electrochemical, opto-electronic and thermal stability, was designed and synthesized in two steps to meet this challenge. By employing PFTPA as dopant-free hole-transport layer in air-processed inverted PSCs, a champion power conversion efficiency (PCE) of up to 16.82% (0.1 cm^2^) was achieved, much superior to that of commercial HTM PEDOT:PSS (13.8%) under the same conditions. Such a superiority is attributed to the well-aligned energy levels, improved morphology, and efficient hole-transporting, as well as hole-extraction characteristics at the perovskite/HTM interface. In particular, these PFTPA-based PSCs fabricated in the air atmosphere maintain a long-term stability of 91% under ambient air conditions for 1000 h. Finally, PFTPA as the dopant-free HTM was also fabricated the slot-die coated perovskite device through the same fabrication condition, and a maximum PCE of 13.84% was obtained. Our study demonstrated that the low-cost and facile homopolymer PFTPA as the dopant-free HTM are potential candidates for large-scale production perovskite solar cell.

## 1. Introduction

Over the past few years, encouraging progress has been made in the field of organic-inorganic hybrid perovskite solar cells [1,2,3,4,5,6,7]. The power conversion efficiency (PCE) of the organic-inorganic halide perovskite solar cells (PSCs) has rocketed to a certified record of 25.8% [8,9], indicating its great potential to compete with traditional silicon solar cells in near future. Substantial effort has been carried out to push the PSC performance to its theoretical limit, including fabrication techniques, device architectures, functional components based on new materials in perovskite layer and charge-transporting layers [10,11,12,13,14]. Two types of device architectures have been widely employed for PSCs: normal (n-i-p) and inverted (p-i-n) configurations, with each type featuring different advantages and challenges. The n-i-p PSC devices currently have superior PCE but typically use transparent electron transport layers (ETLs) of metal oxides that require high-temperature fabrication methods and doped hole transport materials (HTMs) that can introduce device degradation pathways. Moreover, these n-i-p architecture PSCs suffer from a large degree of *J* − *V* hysteresis. As an alternative, the emerging inverted PSCs with a p-i-n architecture use p-type and n-type materials deposited at relatively low temperatures by solution processing as bottom and top charge transport layers, respectively [15,16,17,18]. The inverted PSCs have shown many advantages, such as high efficiencies (as high as >25% using self-assembly hole-extraction layer) [19,20,21,22,23], low-temperature processing on flexible substrates, and, furthermore, negligible *J* − *V* hysteresis effects. Thus, p-i-n PSCs are typically employed in silicon/perovskite [24] and perovskite/perovskite [25] two-terminal tandem devices, which is essentially important for the future of commercial PSC technology.

For inverted PSCs, HTMs not only greatly improve hole extraction and transport from the perovskite layer to electrode, but also have a significant impact on the crystallinities and morphologies of perovskite film, which could boost the PCE and stability of devices [26,27,28]. Especially to achieve superior long-term stability of PSCs, it is necessary to develop efficient dopant-free HTMs. Fundamentally, highly efficient dopant-free HTMs for inverted PSCs have several basic requirements, such as suitable energy levels for perovskite materials, high hole mobility and conductivity, high chemical and thermal stability, excellent film processing ability and film stability [29,30,31]. Since in the inverted devices, HTM is deposited before the perovskite layer, the surface properties of the HTM layer significantly affect the polycrystalline film quality of the perovskite layer. Thus, excellent wettability with perovskite precursor solutions is an essential prerequisite for promoting the crystallization process of perovskite [31,32]. In addition, a weak absorption coefficient in the visible to near-infrared region and high photostability in the ultra-violet region are highly desirable for dopant-free HTMs in p-i-n devices because the light passes through the HTM layer before being absorbed by the perovskite layer [31,32].

Dopant-free polymer HTMs have attracted much attention due to their advantages such as high heat resistance, high hydrophobicity, excellent film-processing ability, and compatibility with the scale roll-to-roll printing technique [32,33]. More importantly, the amorphous nature and strong intrachain charge transfer along the conjugated backbone result in a good balance between high mobility and good film quality, rather than a trade-off for small-molecule HTMs. Although plenty of efficient dopant-free D-A copolymeric HTMs have been reported [34,35,36], most of them were utilized in n-i-p device stacks. Except for their high costs, D-A copolymers also absorb some sunlight in visible region when they are applied to the p-i-n PSCs. It should be noted that the majority of highly efficient PSCs based on D-A type copolymers are fabricated inside a glove box filled with costly inert gas to avoid moisture, which is incompatible with the low-cost and large-scale manufacturing of PSCs in ambient conditions.

The state-of-the-art p-i-n device architectures use fullerene or related derivatives as ETLs and dopant-free polymeric HTMs such as poly[bis(4-phenyl)(2,3,6-trimethylphenyl)amine] (PTAA). However, its low hole transporting ability and high hydrophobicity require additional dopants and wetting treatment for PTAA, which unavoidably reduce the device performance reproducibility [37]. In the case of PEDOT:PSS, a widely used classic water-soluble conducting polymer HTL in inverted PSCs, its acidic and hygroscopic nature can degrade the perovskite layer and corrode anodes, reducing the stability of the solar cells [38]. Furthermore, the mismatched energy level between the valence band of perovskite materials and the work function of PEDOT:PSS could lead to severe non-radiative recombination, limiting the V_OC_ as well as the photovoltaic performance. To date, the inverted PSCs using pristine PEDOT:PSS as the HTL achieved a PCE of 15.05% [39], which is far less than the other dopant-free polymeric HTL, such as PTAA, PII2T8T and poly[3-(4-carboxybutyl)-thiophene-2,5-diyl] (P3CT) [40].

On the other hand, most of the reported highly efficient PSCs were fabricated in a well-controlled glovebox free from moisture and oxygen. Large-scale manufacturing in ambient processing conditions remain challenging both in lab and in factory, because polycrystalline perovskite materials are extremely sensitive to moisture and oxygen in ambient air. Several recent advances in the development of air-processed PSCs mainly focus on the control of the processing of perovskite materials. But little attention has been paid to dopant-free polymeric HTMs in the inverted PSC devices fabricated in air. Our aim is to develop low-cost, highly efficient dopant-free polymeric HTM, which is suitable for air-processed PSCs.

**Scheme 1:**
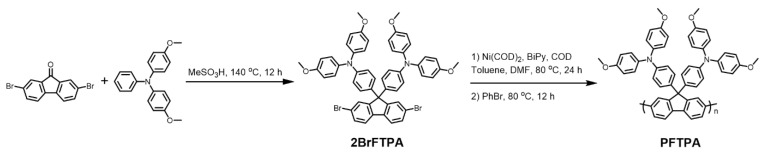
The chemical structure and synthetic route of PFTPA.

Herein, we introduce a novel homopolymer, poly(2,7-(9,9-bis(N,N-di-p-methoxylphenyl amine)-4-phenyl))-fluorene (denoted as PFTPA, see in Scheme 1). PFTPA exhibits matched energy alignment with adjacent perovskite, superior hydrophobicity, and high hole mobility. As a preliminary result, the dopant-free PFTPA-based air-processed p-i-n PSCs exhibit a champion power conversion efficiency (PCE) of 16.82% (0.1 cm^2^) under a 100 mW cm^2^ AM1.5G solar illumination and maintain a long-term stability of 91% under ambient air conditions for 1000 h. These results suggest the great potential of homopolymer PFTPA HTMs for future low-cost large-scale and flexible PSCs application.

## 2. Materials and Methods

### 2.1. Synthesis of PFTPA

#### 2.1.1. Synthesis of 4,4′-(2,7-Dibromo-9H-fluorene-9,9-diyl)bis(N,N-bis(4-methoxyphenyl)aniline) (2BrFTPA)

In a two-necked round bottom flask (50 mL), 2,7-dibromofluorenone (1.50 g, 4.40 mmol), 4,4-dimethoxytriphenylamine (10.85 g, 35.5 mmol) and methylsulfonic acid (4 mL) were mixed. The mixture was heated to 140 °C and kept refluxing for 12 h. Then, the reaction system was cooled to room temperature and the solidified raw products were dissolved with dichloromethane. This mixture solution was washed by saturated saline in turn. The organic phase was dried with anhydrous sodium sulfate before dichloromethane was removed. The crude product was further purified by silica gel column chromatography and recrystallized from a mixed solvent of ethyl acetate/hexane to obtain 2BrFTPA as a white solid (4.10 g, yield 75%). ^1^H NMR (400 MHz, CDCl_3_) δ = 7.53 (d, *J* = 8.1 Hz, 2H), 7.49–7.41 (m, 4H), 7.03 (d, *J* = 8.8 Hz, 8H), 6.90 (d, *J* = 8.8 Hz, 4H), 6.79 (d, *J* = 9.0 Hz, 8H), 6.74 (d, *J* = 8.7 Hz, 4H), 3.76 (s, 12H) ppm. ^13^C NMR (101 MHz, CDCl_3_) δ = 156.06, 153.96, 147.54, 140.73, 138.01, 135.69, 130.72, 129.45, 128.60, 127.00, 121.78, 121.56, 119.67, 114.77, 64.55, 55.57 ppm. MALDI-TOF-MS (*m*/*z*): Calculated for C_53_H_42_Br_2_N_2_O_4_: 928.15; Found: 928.11 [M]^+^.

#### 2.1.2. Polymerization of PFTPA

Bis-(1,5-cyclooctadiene) nickel (130 mg, 0.48 mmol), 2,2-Bipyridine (76 mg, 0.48 mmol), 1,5-cyclooctadiene (52 mg, 0.48 mmol) and anhydrous DMF (5 mL) were added into a 25 mL thick pressure-resistant Schlenk reaction tube, heated to 80 °C under the protection of nitrogen and stirred for 30 min. Monomer 2BrFTPA (370 mg, 0.40 mmol) was dissolved in 8 mL of anhydrous toluene, bubbled with nitrogen for 10 min to remove the air in the solution, and then added to the reaction tube. The reaction system was heated to 80 °C and stirred for 24 h. Bromobenzene (2 mL) was added for end capping and stirring continued at 80 °C for 12 h; then, it was dropped into the mixed solution of methanol/hydrochloric acid (*v*/*v* = 2/1). The catalyst was then filtered and removed. The filter cake was redissolved with chloroform (15 mL), dropped into the mixed solution of methanol/acetone (*v*/*v* = 4/1), and reprecipitated to obtain the solid crude product. The solid crude product was put into Soxhlet extractor, extracted successively with petroleum ether and dichloromethane, and yellow green PFTPA film (260 mg, yield 70%) was formed on the bottle wall after removing dichloromethane by rotary evaporation. ^1^H NMR (400 MHz, CDCl_3_) δ = 7.76 (m, 2H), 7.61 (m, 4H), 7.07 (m, 4H), 6.94 (m, 8H), 6.75 (m, 12H), 3.72 (s, 12H) ppm. GPC (THF, polystyrene standard, 35 °C): M_n_ = 12.2 kDa, M_w_ = 29.9 kDa, PDI = 2.46.

### 2.2. Device Fabrication and Measurement

Materials: A modified PEDOT:PSS solution was prepared by mixing 1 mL of PEDOT:PSS (HC Starck, Baytron P AI 4083), 60 mg of sodium polystyrene sulfonate (molecular weight ~70,000, Sigma-Aldrich, Shanghai, China), and 5 mL of deionized water and stirring for 10 min. A CH_3_NH_3_PbI_3_ precursor solution was prepared by dissolving a 1.2 M PbI_2_ (Sigma-Aldrich, Burlington, MA, USA) and a 1.2 M CH_3_NH_3_I (Greatcell Solar Materials, Beijing, China) in dimethylformamide (DMF) and stirring at 70 °C for 30 min. After cooling to room temperature, solid NH_4_Cl was added to the solution with a concentration of 0–20 mg/mL, and the solution was then stirred for 30 min at room temperature. PFTPA was dissolved in toluene and a 0.5 mg/mL solution was prepared for standby.

Device Fabrication: Patterned ITO glass was cleaned in detergent (Deconex 12PA detergent solution), deionized water, acetone, and isopropanol sequentially by ultrasonication and then treated with UV-ozone for 15 min. For control devices, PEDOT:PSS was dropped onto the ITO glass substrate through a syringe filter (0.2 μm RC filter) and spin coated at 5000 rpm for 20 s. For target devices, PFTPA in toluene was deposited on ITO by spin coating at 5000 rpm for 30 s. The substrate was then heated on a hotplate at 150 °C for 10 min in air. After cooling to room temperature, the substrate was put on a piece of Halyard TERI Wiper. A total of 20 μL of CH_3_NH_3_PbI_3_ solution was dropped on the substrate. Then, a N_2_ gas flow was applied to the substrate from a plastic tube with an inner diameter of 4 mm. The tube was perpendicular to the substrate and the outlet of the tube was 1 cm above the substrate. The flow rate of the N_2_ gas was adjusted using a flowmeter. The solution spread on the substrate and the superfluous solution flowed off the substrate while blowing. The color of the substrate changed from yellow to dark brown in 10 s. Then, the tube was moved around the substrate to dry the film at the edge. Next, the substrate was heated at 100 °C for 30 s. PC_61_BM in chlorobenzene (20 mg mL^−1^) and PEIE were spin coated onto the CH_3_NH_3_PbI_3_ layer at 1000 rpm for 30 s. Finally, a 100 nm Ag was evaporated onto the BCP layer through a shadow mask to produce an active area of 0.1 cm^2^ [33,38].

For the perovskite solar cells made using slot-die coating, ITO glass was cleaned and PFTPA was spin-coated on the substrate as described above. A 0.65 M CH3NH3PbI3 solution with a 10 mg/mL NH_4_Cl additive was coated onto the modified PFTPA layer using a slot-die coater with a setting speed of 8 mm/s. The temperature of the printer bed was set to 60 °C. N_2_ gas with a flow rate of 20 L/min was used to dry the film while coating. PC61BM, PEIE, and Ag layers were deposited as described above.

Device Characterizations: The optical absorption of the perovskite samples was measured using a UV-vis spectrophotometer (Shimadzu UV-1750, Kyoto, Japan). The steady-state photoluminescence (PL) spectra were obtained using a PL microscopic spectrometer (HITACHI, F4600, Tokyo, Japan). The time-resolved photoluminescence (TRPL) was measured at 780 nm using excitation with a 510 nm light pulse from Edinburgh FLS980. The photocurrent density-voltage curves of the perovskite solar cells were measured using a solar simulator (Oriel 94023A, 300 W) and a Keithley 2400 source meter. The intensity (100 mW/cm^2^) was calibrated using a standard Si solar cell (Oriel, VLSI standards). All the devices were tested under AM 1.5G sun light (100 mW/cm^2^) using a metal mask of 0.1 cm^2^ with a scan rate of 10 mV/s.

## 3. Results and Discussion

### 3.1. Synthesis and Design Principle

The chemical structure and synthetic route toward PFTPA are shown in Scheme 1. The design of PFTPA was inspired by 2,2′,7,7′-tetrakis(N,N′-di-p-methoxyphenylamine)-9,9′-spirobifluorene (Spiro-OMeTAD), the most explored HTM. Spiro-OMeTAD contains a spirobifluorene core and four diphenylamine end groups. Its twisted structure ensures high solubility and facilitates the process of the HTM film, but leads to large intermolecular distance and weak intermolecular interaction resulting in its low charge mobility. On the other hand, Spiro-OMeTAD possesses a wide bandgap and a deeper HOMO energy level than many other reported dopant-free polymeric HTMs. Therefore, PFTPA was successively designed by reducing the rigidity of the spirobifluorene core to 9,9-diphenyl-fluorene and extending the conjugated length from one spirobifluorene unit to polyfluorene. The key monomer of 2BrFTPA was obtained by refluxing 2,7-dibromofluorenone and 4,4-dimethoxytriphenylamine (OMeTPA) in methylsulfonic acid without any expensive catalyst with a high isolated yield. Eventually, the homopolymer, PFTPA, was obtained by Yamamoto polymerization according to monomer 2BrFTPA. Detailed synthetic procedures and structural characterizations of monomer and PFTPA were described in the Experimental Section. The chemical structure of 2BrFTPA was confirmed by ^1^H NMR, ^13^C NMR and MALDI-TOF mass spectrum measurements as shown in Figures S1–S3. The target polymeric HTM was readily soluble in common organic solvents such as chloroform, toluene, and chlorobenzene (CB). The average molecular weights (Mn and Mw) and polydispersity index (PDI) of PFTPA were measured via gel permeation chromatography (GPC). The weight-average molecular weight (M_W_) was 29.9 kDa with a PDI of 2.46. Note that the lab synthesis and purification cost of 2BrFTPA and PFTPA are both very low. For example, the total cost for PFTPA is estimated at 16.74 USD/g (the lab synthesis and purification cost is summarized in Table S1), which is much lower than that of many other reported dopant-free polymeric HTMs and shows a promising scale-up strategy for commercial production.

### 3.2. Thermal, Photophysical and Electrochemical Properties

**Figure 1:**
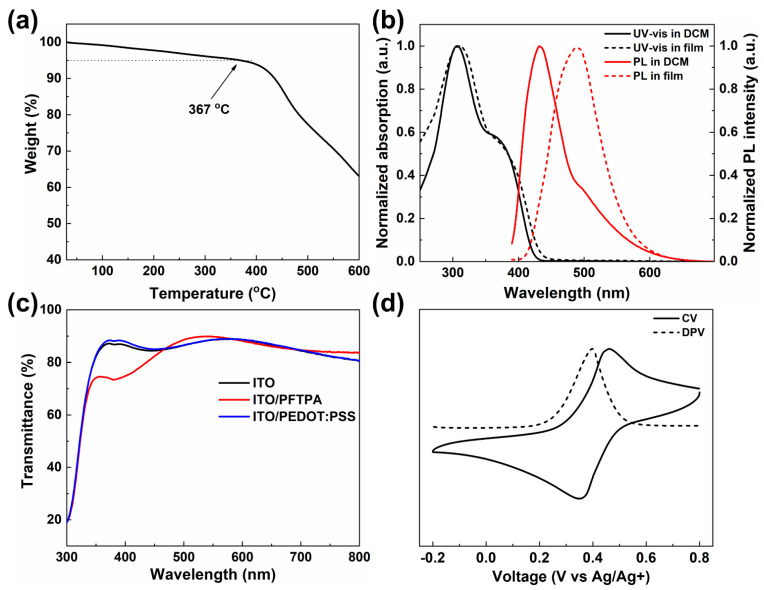
Thermal, photophysical and electrochemical properties of PFTPA. (**a**) TGA curves recorded at a heating rate of 10 °C/min; (**b**) Normalized UV-vis absorption spectra and photoluminescence (PL) spectra of PFTPA in dilute DCM solution (10^−5^ M) and in spin-coated films; (**c**) The transmittance spectra of ITO and ITO with different HTMs; (**d**) The cyclic voltammetry (CV) and differential pulse voltammetry (DPV) curves of PFTPA.

Thermal properties of polymeric HTMs have an important impact on the stability of PSC devices. Thermal properties of PFTPA were characterized via thermogravimetric analyzer (TGA) and differential scanning calorimeter (DSC). As shown in Figure 1a, PFTPA displayed an outstanding thermal stability with decomposition temperatures (*T*_d_, 5% weight loss temperature) of 367 °C. The DSC curves display the temperature-time data of the second heating circle as shown in Figure S5; the glass phase transition temperature (*T*_g_) of PFTPA is about 200 °C. Both TGA and DSC results indicate that PFTPA can be maintained in amorphous states during the thermal annealing process, which is essential for device fabrication and device operation stability.

Normalized ultraviolet visible (UV-Vis) absorption spectra and photoluminescence (PL) spectra of PFTPA in dilute dichloromethane solution (10^−5^ M) and spin-coated thin films are shown in Figure 1b, and the corresponding data are summarized in Table 1. The maximum absorption wavelengths of PFTPA in solution and film state are 307 nm and 310 nm, respectively. The long wavelength absorption edges are estimated to be 421 nm and 434 nm, respectively. The absorption spectra of PFTPA in a dichloromethane solution and thin film are very similar in shape, while the absorption spectrum in film is slightly broadened compared to the spectrum in the solution, indicating that no strong intermolecular π-π stacking exists. The fluorescence emission peaks of PFTPA in dichloromethane solution and thin film are 431 nm and 489 nm, respectively, and the corresponding Stokes shifts are 9397 cm^−1^ and 11,808 cm^−1^, respectively. A larger Stokes shift will benefit the hole-injection of HTMs and the efficiency of PSC devices. According to the initial absorption wavelength of the absorption spectrum of PFTPA in dilute dichloromethane solution (*λ*_Edge_), the optical band gap of PFTPA is calculated at 2.95 eV by equation $E_{g}^{\mathrm{opt}}$ = 1240/*λ*_edge_. The absorbance of PFTPA as a wide bandgap homopolymer is mainly located in the near ultraviolet light region, and PFTPA displays strong blue emission. Thus, in p-i-n PSC devices, the competitive absorption of solar light by the hole transport layer and perovskite layer can be efficiently avoided. Meanwhile, by converting UV light to blue emission, PFTPA could reduce the damage of UV irradiation to perovskite layer and increase the solar light density in the visible region, leading to efficient absorption and conversion of solar light by PSC devices.

The transmittance of the hole transport layer in inverted PSC devices significantly affects the utilization efficiency of sunlight by the perovskite active layer. The light transmission spectra of bare ITO substrate, ITO/PFTPA and ITO/PEDOT:PSS films were measured (shown in Figure 1c). PFTPA has obvious light absorption in the range of 350–450 nm, resulting in low light transmittance in this range. However, at the wavelength ranging from 470 to 800 nm, its light transmittance is better than that of PEDOT:PSS, ensuring more sunlight can efficiently reach the perovskite active layer. Moreover, as discussed above, the absorbed sunlight in the range of 350–450 nm can be partially down-converted to the blue emission of PFTPA, which will also be absorbed by the perovskite layer.

Electrochemical cyclic voltammetry (CV) and differential pulse voltammetry (DPV) were performed to determine the frontier orbital energy levels of PFTPA experimentally. Figure 1d depicts the CV and DPV profile of PFTPA in a dilute DCM solution. As can be seen, PFTPA showed a reversible oxidation process in the positive range. The onset oxidation potential ($E_{\mathrm{onset}}^{\mathrm{OX}}$) of PFTPA is estimated to be 0.34 V (vs. Ag/Ag^+^) with ferrocene as the reference and the oxidation potential value of ferrocene of 0.10 V (vs. Ag/Ag^+^, see in Figure S6). Usually, the HOMO energy level is calculated according to the following Formula (1):

(1) $$HOMO=-\left(E_{\mathrm{onset}}^{\mathrm{OX}}-E_{Fc/Fc+}+5.1\right)\mathrm{eV}.$$

In the end, the HOMO energy level of PFTPA is calculated to be −5.34 eV, which is deeper than that of spiro-OMeTAD (−5.01 eV) and PTAA (−5.1 eV). This value matches the VB energy level (5.40 eV) of MAPbI_3_ well, which will benefit the efficient extraction of the hole at the interface between the hole transport layer and the perovskite active layer. In addition, the deeper HOMO levels of HTMs could benefit the higher open-circuit voltage (V_OC_) of the PSC devices. The LUMO energy level of PFTPA is calculated to be −2.39 eV by adding its optical band-gap energy (*E*_g_ = 2.95 eV) to its HOMO energy. Obviously, PFTPA with much higher LUMO energy level than the conduction band (CB) energy level of MAPbI_3_ (−3.9 eV) could block electron flow into perovskite more effectively.

### 3.3. Density Functional Theory Simulation of PFTPA

**Figure 2:**
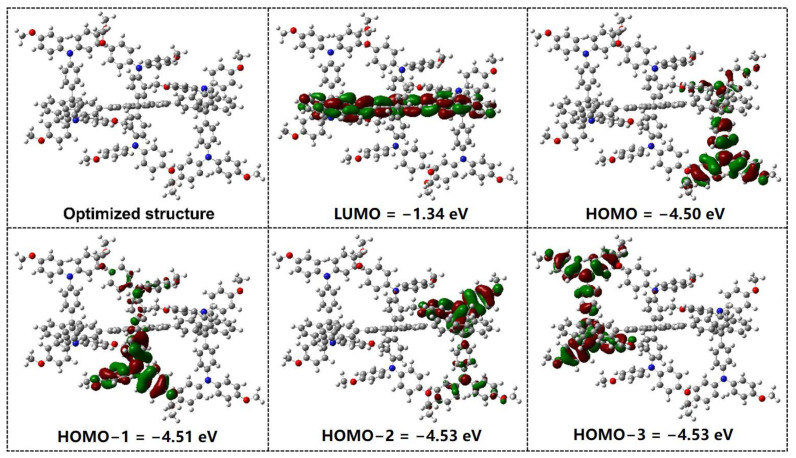
The optimized chemical structure and frontier orbitals distribution of PFTPA.

To further understand the molecular configuration and electron distribution of its frontier molecular orbitals of PFTPA, density functional theory (DFT) simulation was performed using Gaussian 09 at the B3LYP/6-31G(d,p) base set. In order to investigate the twist angle of monomers in conjugated backbone of PFTPA, a model polymer with three repeating units was adopted for theoretical simulation as shown in Figure 2. The optimized geometry shows that the dihedral angles between the middle fluorene unit and the left and right monomers are +33.74° and −33.72°, respectively. This implies that all the 4,4-dimethoxytriphenylamine groups are arranged in a helical manner around the polyfluorene backbone. Thus, these 4,4-dimethoxytriphenylamine groups suppress the π-π stacking between polyfluorene backbone, which explains the similar absorbance spectra of PFTPA in solution and film state. The LUMO orbital of PFTPA is distributed in the polyfluorene backbone, while the HOMO orbital is mainly located around the 4,4-dimethoxytriphenylamine groups in one repeat unit. By plotting the molecular orbits of HOMO-1, HOMO-2 and HOMO-3, all these HOMO orbitals are distributed in one 4,4-dimethoxytriphenylamine group due to the degeneracy of energy levels among the monomers in PFTPA.

The electronic transition process and absorption spectrum of PFTPA in the dichloro-methane solution was also theoretically simulated based on the time-dependent DFT (TD-DFT) method, as shown in Figure S7 and Table S2. The absorption peaks of PFTPA mainly originate from n-π* and π-π* transitions between 4,4-dimethoxytriphenylamine and the polyfluorene backbone. For example, the maximum absorption peak near 310 nm and the absorption shoulder account for the n-π* and π-π* transition absorption, respectively.

### 3.4. Photovoltaic Performance of the PFTPA-Based PSC Devices

**Figure 3:**
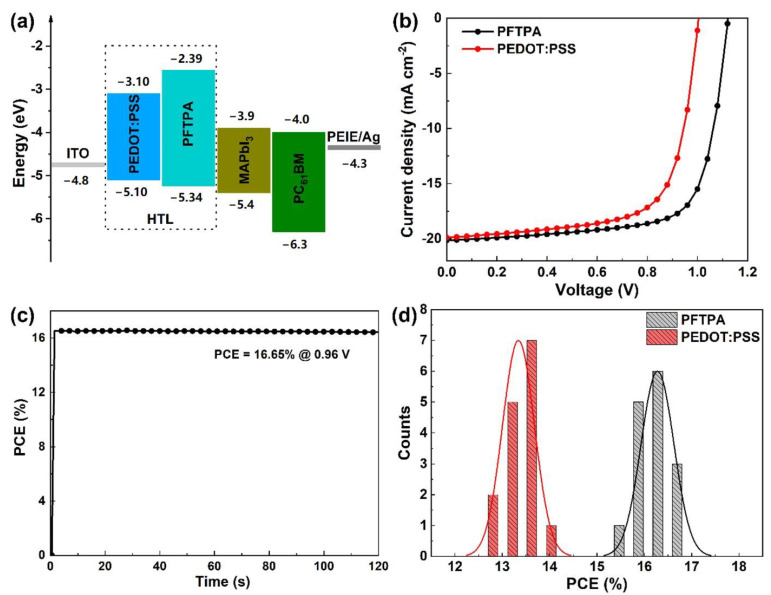
(**a**) The energy diagram of perovskite solar cells, (**b**) the *J* − *V* curves of perovskite solar cells based on HTMs of PFTPA and PEDOT:PSS, (**c**) the stabilized PCE of PFTPA-based PSC device obtained near the maximum power point voltage at 0.96 V, (**d**) histogram of 15 devices based on PFTPA and PEDOT:PSS.

To evaluate its behavior as dopant-free HTM, PFTPA was incorporated without any doping additives in inverted PSCs. PEDOT:PSS was used as HTM for control devices without additives as well. We fabricated an inverted PSC device with the planar configuration of ITO/HTLs/MAPbI_3_/[6,6]-phenyl-C61-butyric acid methyl ester (PC_61_BM)/PEIE/Ag as shown in Figure 3a. The perovskite layer was prepared in air environment by air blowing assisted drop coating (BADC) [41]. PC_61_BM was used as an electron transporting layer, and ethoxylated polyethyleneimine (PEIE) was used as a cathode modification layer to improve charge extraction efficiency. As shown in Figure 3a, the HOMO energy level of PFTPA and the LUMO energy level of PC_61_BM achieved good energy level matching with the valence band and conduction band of MAPbI_3_, respectively, which effectively promotes the extraction and transmission of photogenerated carriers at interfaces.

The *J* − *V* characteristic curves of the optimal perovskite device are shown in Figure 3b. The open-circuit voltage (V_OC_) of the perovskite device based on the PFTPA is 1.13 V, the short-circuit current (J_SC_) is 19.66 mA cm^−2^, the filling factor (FF) is 75.7%, and the PCE reaches 16.82%. In comparison, the PCE of the control device based on PEDOT:PSS under the same preparation conditions is only 13.80% because of the much lower V_OC_ (1.00 V). According to the operating principle of the perovskite solar cells, it can be determined that the V_OC_ of the device is affected by the band gap of the photoactive layer, the HOMO energy level of the hole transporting layer and the LUMO energy level of the electron transporting layer. Therefore, compared with PFTPA, the higher HOMO energy level of PEDOT:PSS leads to the increased energy loss at the hole extraction interface and results in a much lower V_OC_ of the control device. Under a fixed bias voltage (0.96 V), the stable output efficiency of the champion device based on PFTPA is 16.65%, as shown in Figure 3c, which is consistent with the PCE obtained in the *J* − *V* curves. The repeatability of device preparation is an important index to evaluate the preparation method and material performance. The PCEs of 15 perovskite solar cells based on PFTPA and PEDOT:PSS are counted, as shown in Figure 3d. Both of them show a narrow statistical distribution, indicating that the above devices have good repeatability. Additionally, PFTPA as the dopant-free HTM has been applied to the slot-die coated inverted PSCs. As a preliminary result, the slot-die coated inverted PSCs at a device area of 0.1 cm^2^ showed a max PCE of 13.84% with a J_SC_ of 19.50 mA cm^−2^, a V_OC_ of 1.08 V, and an FF of 65.7% (Figure S13 and Table 1). It should be mentioned that as a preliminary result, PCEs of the dopant-free PFTPA-based air-processed p-i-n PSCs maintain a long-term stability of 91% under ambient air conditions for 1000 h (see Figure S8). These results suggest the great potential of homopolymer PFTPA HTMs for future low-cost large-scale and flexible PSCs application.

### 3.5. Morphology Analysis of Perovskite Films

**Figure 4:**
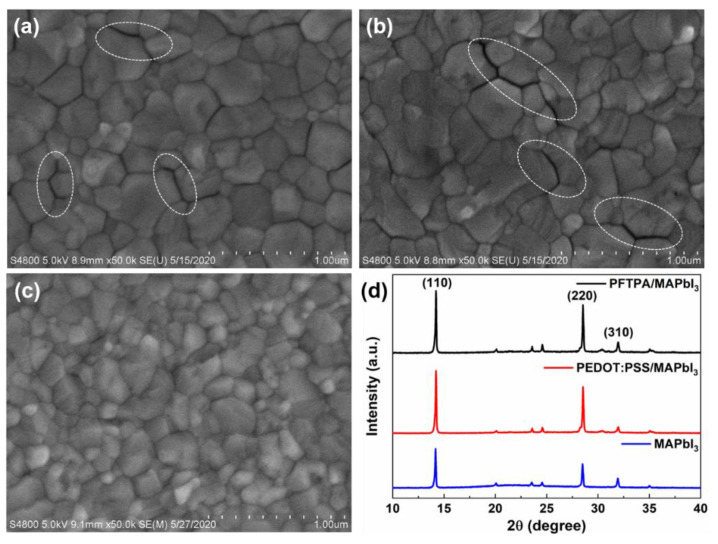
SEM images of perovskite on the top of (**a**) PFTPA, (**b**) PEDOT:PSS and (**c**) ITO substrates, (**d**) the X-ray diffraction patterns of perovskite on different substrates.

The effects of PFTPA and PEDOT:PSS as HTM substrates on the growth quality of perovskite crystals were systematically studied by means of scanning electron microscopy (SEM) and thin film X-ray diffraction (XRD) as shown in Figure 4. Owing to the low solubility of PFTPA in polar solvents, it was possible to fabricate a high-quality perovskite crystalline film on its surface by using the solution-processing method. The contact angle between HTM and DMF was measured (Figure S9). The measured contact angles on ITO, PEDOT:PSS and PFTPA were 9.3°, 18.4° and 77.6°, respectively. This discrepancy in contact angle may result in different morphology of the solution-processed perovskite film. That is, the lesser wettability toward DMF of the PFTPA surface would suppress the heterogeneous nucleation and thus facilitate the grain boundary migration in grain growth, leading to large grain sizes of the resulting polycrystalline perovskite film. To further confirm their good crystal qualities, SEM images of the perovskite films deposited on the top of ITO, PEDOT:PSS or PFTPA substrate were given. The perovskite crystal sizes on PFTPA or PEDOT:PSS-modified ITO substrate were significantly larger (Figure 4a,b) than that on the bare ITO substrate (Figure 4c). The average grain size deduced from SEM images was 158.3 nm, 233. 3 nm and 271.9 nm for perovskite crystals on ITO, PEDOT:PSS and PFTPA, respectively (Figure S10). According to XRD patterns (Figure 4d), three films displayed similar diffraction peaks at 14.12°, 28.36° and 31.81°, assigned to the (110), (220) and (310) planes of perovskite, respectively. The appearance of the intense XRD peaks of the perovskite film deposited on PEDOT:PSS and PFTPA substrates further indicated their good crystallinity. The grain sizes of perovskite crystals on ITO, PEDOT:PSS and PFTPA substrates estimated according to XRD profiles were 163.7 nm, 231.9 nm and 276.5 nm, respectively. The grain size distribution trend based on XRD profiles is consistent with the SEM test results. It should be mentioned that the perovskite crystals grown on PFTPA substrates were more compact and uniform, while the perovskite crystals grown on PEDOT:PSS substrates had more obvious crystal boundary defects (dot-line circling in Figure 4a,b). These defects lead to carrier recombination and non-radiative recombination, which lead to energy loss, reduction in FF and V_OC_ of PSC devices, and ultimate effect on the photovoltaic performance of the devices. At the same time, boundary defects like pinholes provide channels for water vapor infiltration, which can often compromise the performance of PSC devices, especially their stability [42].

The morphological characteristics of perovskite films also include atomic force microscopy (AFM). As shown in Figure S11, the root mean square (RMS) roughness of perovskite films on PFTPA and PEDOT:PSS substrates are 16.22 nm and 17.22 nm, respectively. At the same time, more obvious holes can be observed in the latter. As crystal boundary defects, these holes also lead to the degradation of the performance of PSC devices. The above characterization results consistently show that PFTPA as the hole transport layer is more conducive to the growth of crystalline perovskite films on its surface. In the end, PSC devices based on PFTPA hole-transporting layer show higher FF and PCE.

### 3.6. SCLC Measurements of PFTPA for Its Hole-Transporting Morbility

In order to further verify the hole-transporting characteristics of polymeric HTM PFTPA, a hole-only device based on PFTPA was fabricated and tested. The device structure is ITO/PEDOT:PSS/PFTPA/MoO_3_/Ag. Space charge limited current (SCLC) method was used for this measurement. The *J* − *V* characteristic curve of the hole-only device is shown in Figure S12. The fitting calculation of hole mobility of PFTPA (*µ_h_*) was carried out according to the simplified Mott-Gurney formula:

(2) $$J=\frac{9}{8}\varepsilon_{0}\varepsilon_{r}\mu_{h}\frac{V^{2}}{L^{3}},$$

where *J* is the current density, *ε*_0_ represents the vacuum permittivity, and *ε_r_* is the relative dielectric constant (assuming *ε_r_* = 3 for organic materials) [43], *V* represents the bias voltage applied to the device, *L* represents the thickness of the hole transport layer (about 80 nm), *μ_h_* is the hole mobility. The calculated hole mobility of PFTPA is 1.12 × 10^−5^ cm^2^ V^−1^ s^−1^, which is higher than the reported hole mobility of PEDOT:PSS of 6.86 × 10^−6^ cm^2^ V^−1^ s^−1^ [44]. Based on the above characterization results, PFTPA benefits from its unique 4,4-dimethoxytriphenylamine helical side group structure to enhance the carrier transport efficiency between molecules, which makes it show more efficient carrier transport capacity than PEDOT:PSS, and the corresponding perovskite cell devices achieve higher photovoltaic performance.

### 3.7. Steady-State PL and TRPL Measurements

**Figure 5:**
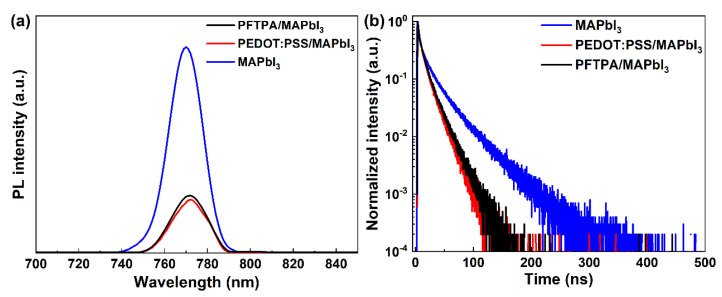
(**a**) The steady-state PL spectra and (**b**) time-resolved PL (TRPL) spectra of the HTMs/MAPbI_3_ films with excitation at 510 nm.

Steady-state photoluminescence (PL) and time-resolved photoluminescence (TRPL) of the HTMs/MAPbI_3_ films were conducted to further analyze and understand the charge extraction and transmission process at the hole transport layer/perovskite interface, as shown in Figure 5. For steady-state PL spectra, the quenching of luminescence intensity indicates the efficient separation of photogenerated carriers, so the carrier extraction efficiency at the interface can be qualitatively analyzed. Based on the above principle, the area integral of the steady-state PL spectrum emission peak was calculated. The photoluminescence intensity of PFTPA/MAPbI_3_ and PEDOT:PSS/MAPbI_3_ thin films decreased to 26.3% and 25.4% of that of pure MAPbI_3_ thin films, respectively, indicating that both PFTPA and PEDOT:PSS showed high carrier extraction efficiency as hole transport materials. The photoluminescence lifetime of the hole transport material/perovskite films was characterized by TRPL spectra. The shorter the lifetime, the more efficient the hole transport process at the interface. As shown in Figure 5b, the TRPL spectrum is excited by a 510 nm wavelength and collected at a 770 nm wavelength. Both perovskite single-component films and HTM/perovskite bilayer films have a fast and a slow decay process, which are fitted by the following double exponential formula, and then the average luminescence lifetime (*τ_avg_*) is calculated:

(3) $$I\left(t\right)=A_{1}\exp\left(-\frac{t}{\tau_{1}}\right)+A_{2}\exp\left(-\frac{t}{\tau_{2}}\right)+A_{0},$$

(4) $$\tau_{avg}=\frac{A_{1}\tau_{1}^{2}+A_{2}\tau_{2}^{2}}{A_{1}\tau_{1}+A_{2}\tau_{2}}.$$

Fast decay luminescence lifetime of perovskite single-component films (*τ*_1_) and slow decay luminescence lifetime (*τ*_2_) are 3.1 ns and 24.8 ns, respectively. The average luminescence lifetime (*τ_avg_*) of a perovskite single-component film is 21.4 ns. For a PEDOT:PSS/MAPbI_3_ bilayer film, τ_1_ and τ_2_ are 2.5 ns and 13.0 ns, respectively, with a *τ_avg_* of 11.4 ns. The τ_1_ and τ_2_ of PFTPA/MAPbI_3_ bilayer films are 2.8 ns and 14.5 ns, respectively, and PFTPA/MAPbI_3_ bilayer films display a *τ_avg_* of 12.5 ns. Compared with pure perovskite films, the luminescence lifetime of the hole transport layer decreases significantly, that is, the photogenerated hole transport process is more efficient. Meanwhile, PEDOT:PSS/MAPbI_3_ bilayer films have shorter luminescence lifetime, which indicates that the charge transfer process at the interface is more efficient. The above steady-state PL and TRPL test results show that PEDOT:PSS, as a hole transport material, is slightly better than PFTPA in hole extraction and transport. However, the performance test results of perovskite solar cells prepared in this chapter show that the perovskite solar cells based on PFTPA have better photovoltaic performance, which may be related to the good film-forming property of PFTPA, the improvement of perovskite crystal growth quality and the more matching energy level structure.

## 4. Conclusions

In summary, a dopant-free polymeric HTM PFTPA was synthesized by a simple and efficient two-step method. The polymer takes polyfluorene as the main chain backbone and 4,4-dimethoxytriphenylamine as the side chain. Its hole mobility reaches 1.12 × 10^−5^ cm^2^ V^−1^ s^−1^. When PFTPA is applied to inverted planar perovskite solar cells, its good film-forming property promotes the growth of MAPbI_3_ films with high crystallinity. At the same time, due to its matching HOMO energy level, the V_OC_ of the champion device reaches 1.13 V, and the PCE becomes 16.82%, which is much higher than the control device using PEDOT:PSS as HTM (PCE = 13.80%). This work provides a new idea for the design and synthesis of dopant-free polymeric HTMs. In terms of device preparation methods, the preparation of HTLs and a perovskite photoactive layer of devices are completed, performed in an air environment, with lower equipment requirements and manufacturing costs. This method is of great significance for the development of low-cost large-size perovskite solar cell devices.

## Figures and Tables

**Table 1:** The photovoltaic performance of perovskite solar cells.

	Scan Direction	V_OC_ (V)	J_SC_ (mA cm^−2^)	FF (%)	PCE (%) ^a^
PFTPA	Reverse	1.13	19.66	75.7	16.82
	Forward	1.12	20.08	72.5	16.30
Slot-die	Reverse	1.08	19.50	65.7	13.84
PEDOT:PSS	Reverse	1.00	19.87	69.5	13.80

Note: ^a^, average value for PCE device according to 15 devices.

## Data Availability

The data presented in this study are available on request from the corresponding author.

## Supplementary Materials

The following supporting information can be downloaded at: https://www.mdpi.com/article/10.3390/polym15122750/s1. Figure S1: ^1^H NMR spectrum of 2BrFTPA in CDCl_3_; Figure S2: ^13^C NMR spectrum of 2BrFTPA in CDCl_3_; Figure S3: MALDI-TOF MS spectrum of 2BrFTPA; Figure S4: ^1^H NMR spectrum of PFTPA in CDCl_3_; Figure S5: DSC curves of PFTPA recorded at a heating rate of 10 °C/min and a cooling rate of 20 °C/min; Figure S6: The cyclic voltammetry (CV) curve of Ferrocene measured with Ag/Ag^+^ as the reference electrode in acetonitrile; Figure S7: The overlay of TD-DFT simulation and experimental UV-vis spectra of PFTPA; Figure S8: The longtime device stability of PFTPA-based PSC; Figure S9: The measured contact angles of DMF (a) on ITO substrate, (b) on PEDOT:PSS substrate and on PFTPA substrate; Figure S10: The perovskite crystal size distribution; Figure S11: AFM height images (size: 5 μm × 5 μm) of perovskite on (a) PFTPA and (b) PEDOT:PSS substrates; Figure S12: The *J* − *V* curve of hole-only device based on PFTPA; Figure S13: The *J* − *V* curve of slot-die coated perovskite device based on PFTPA; Table S1: The synthetic cost analysis of PFTPA in this study; Table S2: The calculation results of TD-DFT.
