# Supplementary material for: Dopant-Free Hole-Transporting Material Based on Poly(2,7-(9,9-bis(N,N-di-p-methoxylphenylamine)-4-phenyl))-fluorene for High-Performance Air-Processed Inverted Perovskite Solar Cells
Source: Polymers (Basel). 2023 Jun 20;15(12):2750. doi: 10.3390/polym15122750 (PMC10301706; doi:10.3390/polym15122750)
Supplement: Supplementary file 1 [file polymers-15-02750-s001.zip › polymers-2436730-supplementary.pdf]

Supplementary Information

Dopant-Free Hole-Transporting Material Based on Poly(2,7-(9,9-bis(*N,N-di-p*-Methoxylphenylamine)-4-phenyl))Fluorene for High-Performance Air-processed Inverted Perovskite Solar Cells

Baomin Zhao [a,*], Meng Tian [a], Xingsheng Chu [a], Peng Xu [a], Jie Yao [a], Pingping Hou [b], Zhaoning Li [a] and Hongyan Huang [b,*]

[a] State Key Laboratory for Organic Electronics and Information Displays &Jiangsu Key Laboratory for Biosensors, Institute of Advanced Materials (IAM), Nanjing University of Posts and Telecommunications, 9 Wenyuan Road, Nanjing 210023, Jiangsu, China

[b] School of Electronic Information, Nanjing Vocational College of Information Technology, 99 Wenyuan Road, Nanjing 210023, Jiangsu, China

Technology, 99 Wenyuan Road, Nanjing 210023, Jiangsu, China
*Corresponding authors. E-mail addresses: provost@nwpu.edu.cn (W. Huang)

NMR and MALDI-Tof spectra

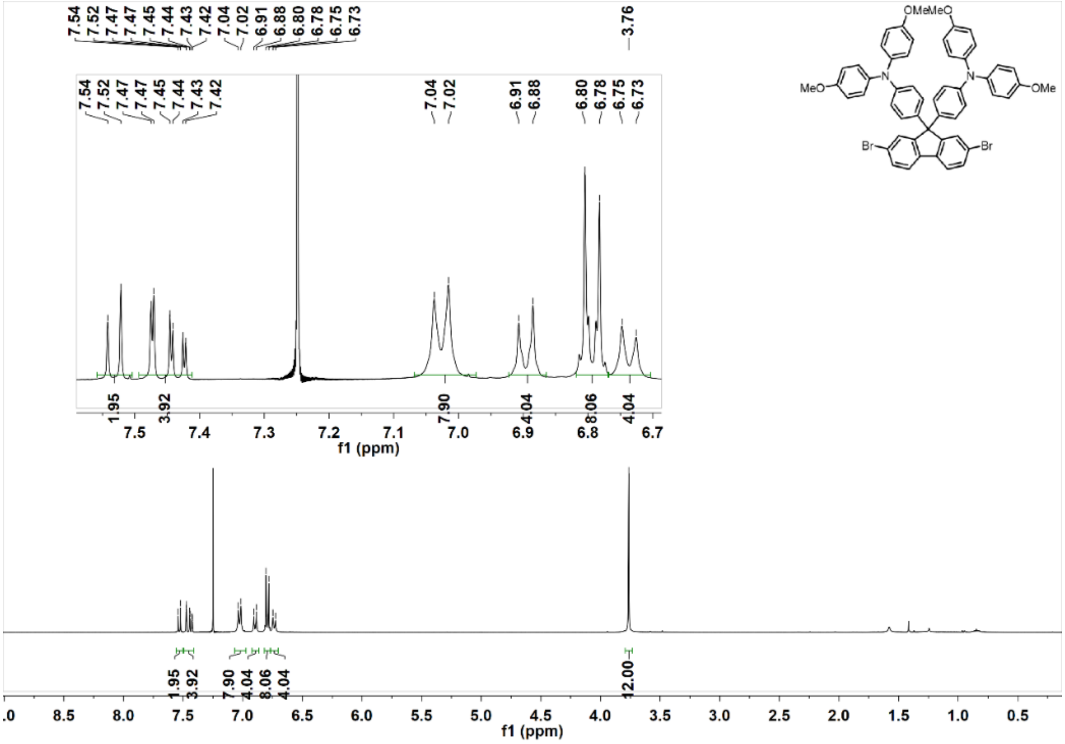

Figure S1 ^{1}H NMR spectrum of 2BrFTPA in $CDCl_3$.

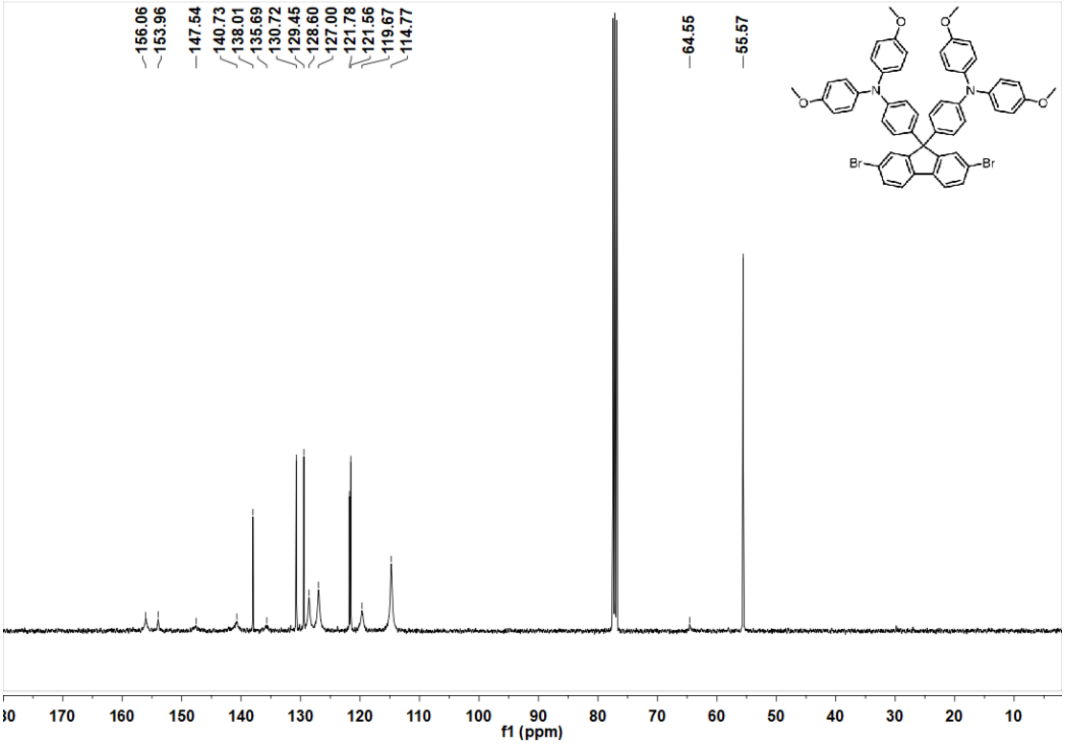

Figure S2 ^{13}C NMR spectrum of 2BrFTPA in $CDCl_3$.

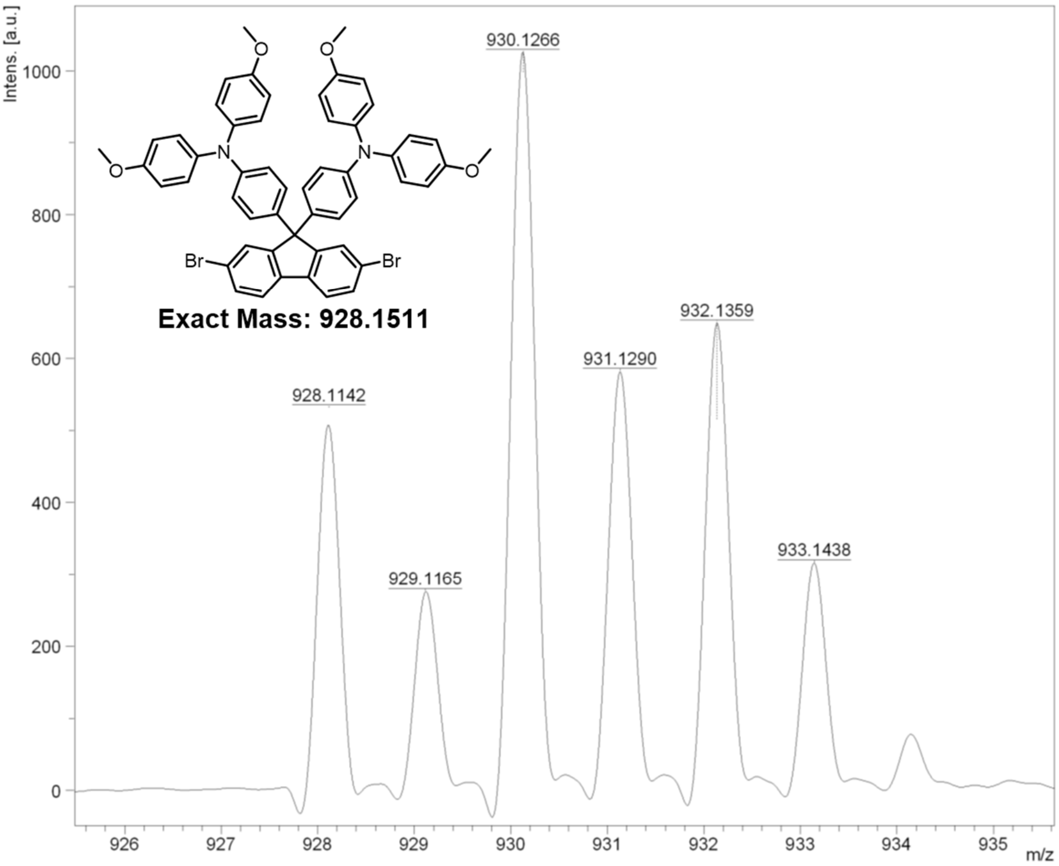

Figure S3 MALDI-TOF MS spectrum of 2BrFTPA.

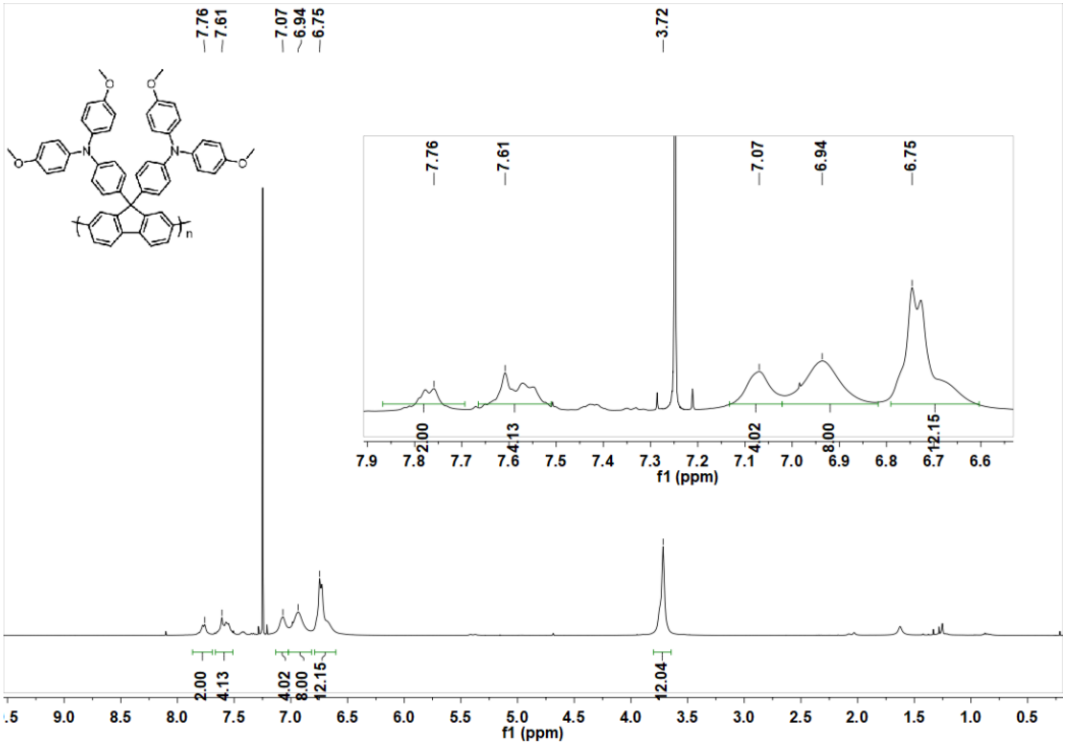

Figure S4 ^{1}H NMR spectrum of PFTPA in $CDCl_3$.

The cost of PFTPA in this study

Table S1 The synthetic cost analysis of PFTPA in this study.

	Reagent	Weight (g or L)	Price ($/g or L) [a]	Cost ($)	Cost ($/g)
2BrFTPA	4,4-dimethoxytriphenylamine	10.85	1.75	18.98	5.35
	2,7-dibromo-9H-fluoren-9-one	1.5	0.36	0.54	
	Methylsulfonic acid	6.0	0.028	0.17	
	Dichloromethane	0.50	1.85	0.93	
	Ethyl acetate	0.3	1.22	0.37	
	hexane	0.80	1.0	0.8	
	Na_2SO_4	30	0.0035	0.105	
PFTPA	2BrFTPA	0.37	5.35	1.98	16.74
	bis-(1,5-cyclooctadiene) nickel	0.13	12.25	1.594	
	2,2-Bipyridine	0.076	0.39	0.03	
	1,5-cyclooctadiene	0.052	0.38	0.02	
	Bromobenzene	3.2	0.05	0.16	
	methanol	0.2	0.85	0.17	
	HCl (3M)	0.1	0.3	0.03	
	Toluene	0.05	1.15	0.0575	
	chloroform	0.02	2.4	0.048	
	acetone	0.25	1.05	0.26	

[a] The price information was obtained from Energy Chemical Technology (Shanghai) Co., Ltd and Shanghai Titan Scientific Co., Ltd.

DSC

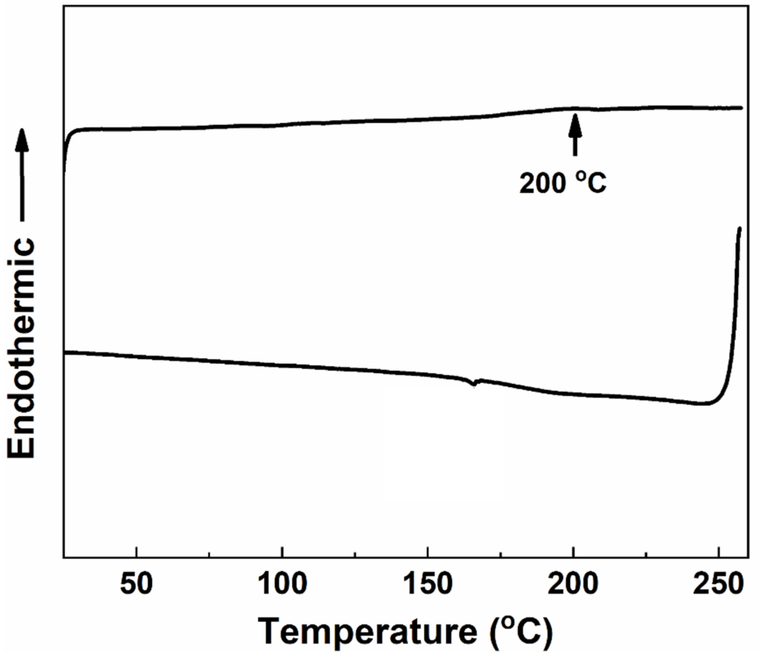

Figure S5 DSC curves of PFTPA recorded at a heating rate of 10 °C/min and a cooling rate of 20 °C/min.

CV curve for Ferrocene vs Ag/Ag$^+$

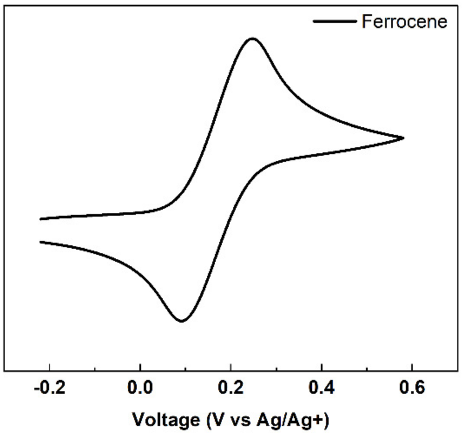

Figure S6 The cyclic voltammetry (CV) curve of Ferrocene measured with Ag/Ag$^+$ as the reference electrode in acetonitrile.

DFT calculation

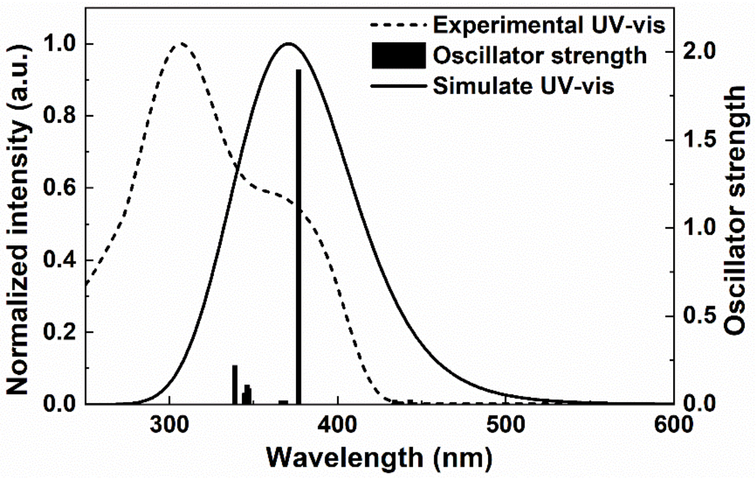

Figure S7 The overlay of TD-DFT simulation and experimental UV-vis spectra of PFTPA.

Table S2 The calculation results of TD-DFT

State	λ/nm	*f*	Major transitions	Character	λ_{exp}/nm
2	338	0.0270	H-3 -> L (49%)<br>H-4 -> L (39%)	n-π*	310
5	376	0.0235	H-3 -> L (39%)<br>H-4 -> L (37%)	π-π*	370

Device stability

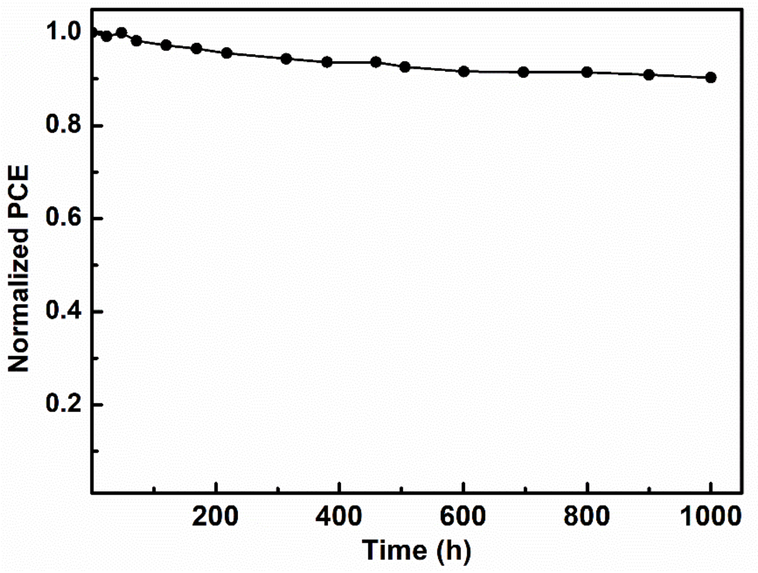

Figure S8 The longtime device stability of PFTPA-based PSC.

The contact angle between HTM and DMF.

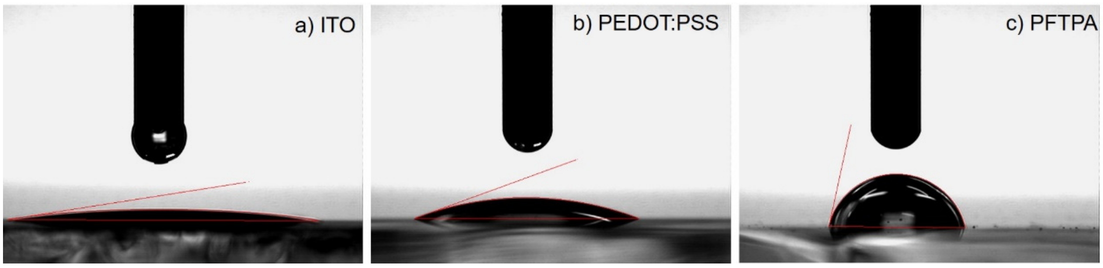

Figure S9. The measured contact angles of DMF a) on ITO substrate, b) on PEDOT:PSS substrate and on PFTPA substrate.

The perovskite crystal size distribution.

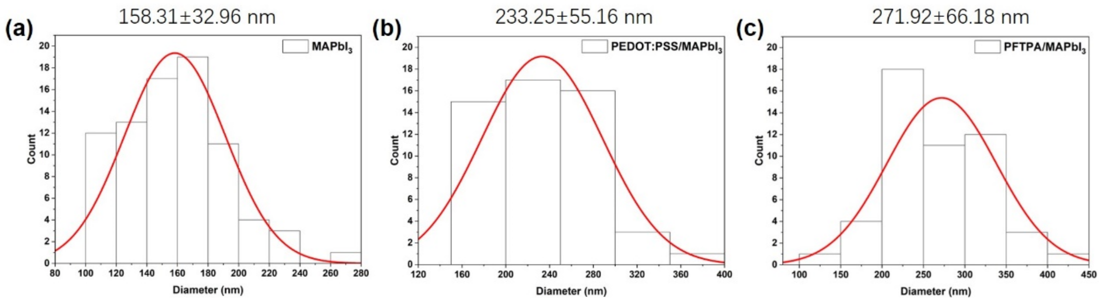

Figure S10. The perovskite crystal size distribution, a) $MAPbI_3$ @ bare ITO substrate, b) MAPbI3@PEDOT:PSS/ITO substrate and c) $MAPbI_3$@PFTPA/ITO substrate, estimated according to the statistical data deduced from the SEM images shown in Figure 4.

AFM

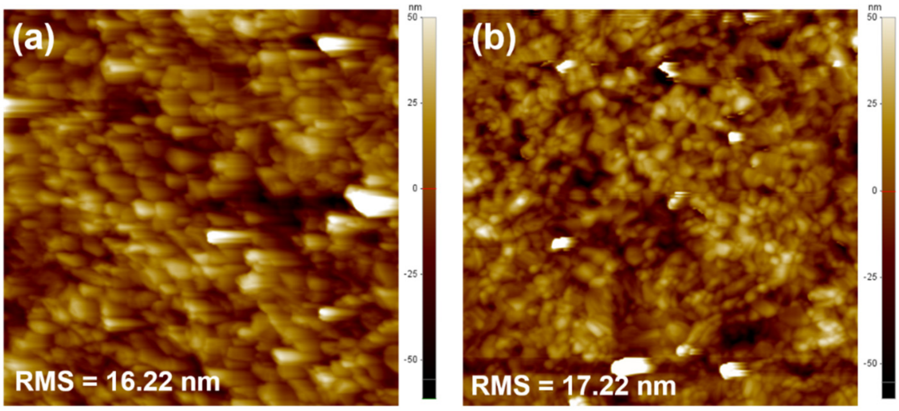

Figure S11. AFM height images (size: 5 μm × 5 μm) of perovskite on (a) PFTPA and (b) PEDOT:PSS substrates.

SCLC

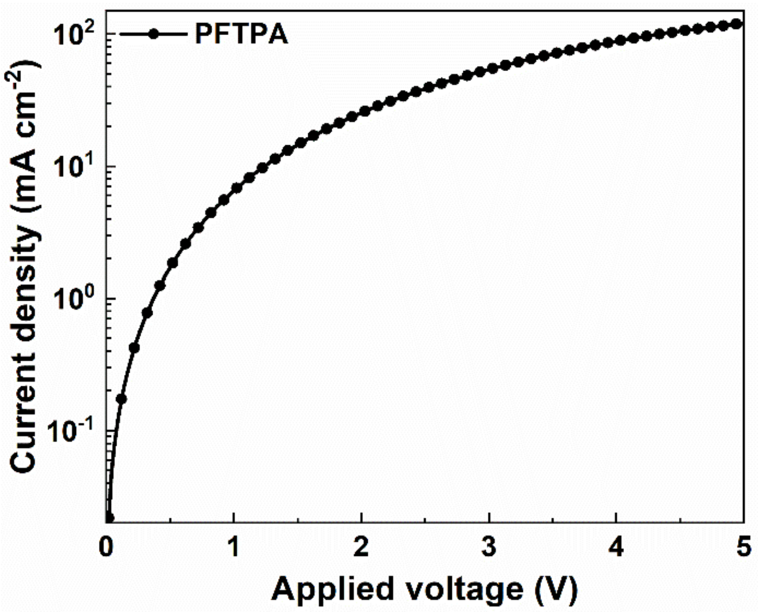

Figure S12. The *J-V* curve of hole-only device based on PFTPA.

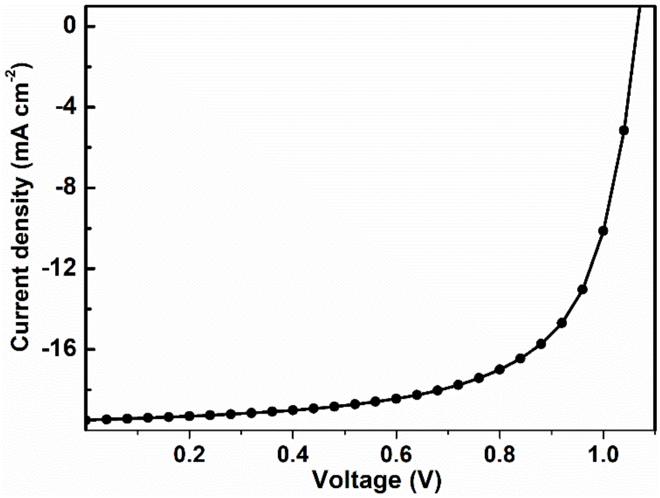

Figure S13. The *J-V* curve of slot-die coated perovskite device based on PFTPA.
